# Glucose measurements in sheep using a long-term continuous glucose monitor designed for humans

**DOI:** 10.3389/fvets.2024.1458044

**Published:** 2024-11-13

**Authors:** Barbara Roqueto dos Reis, Ryan K. Wright, Riley Thompson, Nikki Tabatabai, Katherine Amirault, Sathya Sujani, Robin R. White

**Affiliations:** ^1^White Sand Research Unit, Mississippi State University, Poplarville, MS, United States; ^2^School of Animal Sciences, Virginia Tech, Blacksburg, VA, United States

**Keywords:** sheep, glucose infusion, glucose tolerance test, glucometer, noninvasive glucose measurement

## Abstract

This study evaluated the feasibility of utilizing a continuous glucose monitors (CGM) designed for use in humans to measure glucose levels in sheep. Four Suffolk x Dorset sheep were fitted with jugular catheters and FreeStyle Libre 2 (Abbott®) glucose monitors. Glucose concentration from the CGM were compared with those from a glucometer and traditional assays during a hyperglycemic clamp, aiming to explore a broader range of physiological glucose concentrations in a controlled manner. Measurements were taken every five minutes during the infusion and every ten minutes post-infusion until baseline levels were restored. Relationships were analyzed using a linear mixed-effects model with glucometer readings as the response variables, GCM reading as fixed effects, and animal as random effect with significant level of *p* < 0.05. The results demonstrated a significant linear correlation between the CGM and glucometer readings both during the infusion (*p* = 0.0003) and afterward (*p* = 0.006). A glucose calorimetric test was used to confirm glucose concentrations on samples and used as gold standard. Although the glucometer and CGM did not differ from the assay method, they did differ from one another (*p* = 0.045). Upon more in-depth analysis, the random intercepts for animal were highly significant and one CGM showed values numerically much higher than other CGM and other glucose analysis methods. No difference among methods was observed (*p* = 0.715) when the outlier animal was omitted. While promising, CGM demands confirmation of initial readings and standardization against established methods before wider adoption in research or clinical applications.

## 1 Introduction

In both research and clinical settings involving animals, traditional methods for assessing the glucose-insulin relationship rely on the collection of blood samples. However, these conventional methods require frequent blood sampling to quantify glucose and insulin concentrations which can induce stress in the animals. Continuous glucose monitors (CGMs) have been developed for monitoring glucose levels in diabetic patients and have been available on the market since the early 2000s (1). These devices use glucose oxidase to measure interstitial glucose levels, because the glucose oxidase generates an electrical current that is quantified to estimate blood glucose (2), and the results are converted into glucose levels by proprietary manufacturer algorithms. Currently, there are no commercially available CGM systems specifically designed for animals, but human CGM have been evaluated in cats (3), dogs (4), foals (5), and dairy cows (6).

Despite success in other species, their use has not yet been investigated in sheep, nor has it been expressly investigated during exogenous glucose challenges, as are commonly used in physiological and nutrition research. Exogenous glucose challenges offer the opportunity to monitor an animal’s physiological reaction to various factors (7–9), and successful results rely on proper monitoring of interstitial glucose concentration during these challenges. As an integral part of animal research, glucose challenges are common practice, however they utilize glucose assay kits or glucometers to produce glucose concentration results (10–12). Conversely, while CGM have been tested in animal research settings, they have not been used during an endogenous glucose challenge and therefore exposed to a different range of concentrations. This is true of their use in both livestock (5, 13) as well as in other species such as dogs and cats (4). To assess the true potential of CGM in animal research, this technology must be tested under a range of interstitial glucose concentrations such as those presented during a glucose challenge.

This study aimed to evaluate the feasibility of utilizing a human designed CGM for measuring glucose levels in sheep. To test the device’s capability to detect changes in glucose concentration from normal endogenous processes as well as experimental procedures, a hyperglycemic clamp was performed on the animals. We hypothesized that the CGM would obtain similar readings when compared to a conventional hand-held glucometer and with the standard laboratory essay using plasma from blood samples collected during a hyperglycemic clamp.

## 2 Materials and methods

All procedures and animals in this in this study were approved by the Institutional Animal and Care and Use Committee at Virginia Polytechnic and State University (IACUC #22-138). Four individually housed Suffolk x Dorset ram were used in the study.

Animals were approximately nine months of age and averaged 50 ± 1.81 kg body weight (BW). All animals were fitted with a bilateral jugular catheter and with a FreeStyle Libre 2 system (Abbott Laboratories, Chicago, IL) placed on the animal’s neck after shaving the area and cleaning with alcohol. For better adhesion, superglue was added to the transmitter adhesive patch prior to application. To investigate the CGM sensor’s ability to detect an increase or decrease in glucose concentrations (GC), all animals were subjected to a hyperglycemic clamp. Prior to the glucose infusion (4.58 M, pH 7.36, sterile solution), the basal CG was determined, and the infusion was initiated at 165 mg/kg BW/min, adjusting as necessary to achieve a circulating glucose level that was twice the basal concentration, and stable at this level for 30 min. To achieve the goal of doubling concentrations, glucose was infused for an average of three hours. During the infusion, blood samples were collected every five minutes and measured using a CVS Health Advanced Glucose Meter and CVS Health Advanced Glucose Meter Test Strips (CVS Health Corporation, Woonsocket, RI). At each time point, a corresponding reading from the CGM was obtained by scanning the CGM with the manufacturer’s reading device, attuned such that one reader was aligned with each sensor. When the animals achieved double the basal GC, with a stable plateau where the infusion rate was unchanged and the blood concentration was stable at roughly twice basal GC, the glucose infusion was terminated. After stopping the infusion, blood samples were collected every ten minutes until the animals returned to baseline blood GC. The return to basal GC post-infusion typically took one hour.

During most of the clamp procedure, low-volume blood samples were collected. For these samples, 1 mL of blood was cleared from the catheter to ensure no heparin contamination in sampling. A sample of 1 mL was then obtained, from which a drop was used for the glucometer reading. The remainder of the sample was discarded, and 0.5 mL of 20 unit/mL heparinized saline was pushed to hold the catheter. To allow for glucometer and CGM comparison with traditional benchtop assay measures of glucose concentrations, high-volume blood samples were collected at baseline (*n* = 1) and during the glucose plateau phase (*n* = 6). To collect these high-volume samples, 1 mL of blood was cleared from the catheter to minimize contamination, after which a 5 mL sample was collected, using a 10 mL sodium heparin vacutainer (Becton Dickinson, Franklin Lakes, NJ). Similarly to the approach with low-volume samples, 0.5 mL of heparinized saline was injected post-sampling to maintain catheter patency. After completion of all clamps, full-volume samples were thawed and plasma was isolated by centrifuging at 1.500 × g for 10 min at −4°C. Plasma samples were stored at −20°C until later analyzed for true glucose concentrations in triplicate using the Invitrogen Glucose Colorimetric Detection Kit (Thermo Fisher Scientific Inc., Waltham, MA) following manufacturer instructions.

### 2.1 Statistical analysis

Statistical analyses were conducted using R Statistical Software v 4.1.2 (R Core team, 2021). Models were derived using the lme4 package (14) and estimated marginal means were compared using the emmeans package (15). To compare the results of the glucometer and CGM, both of which had readings every five to ten minutes, the first two models were developed. Relationships between the measurement approaches were analyzed as a linear mixed effect model. The response variable was the readings from the handheld glucometer (ground truth observations). The model fitted was as follows:


$$Y_{ij}=\mu +\alpha_{i}+\beta_{j}+\gamma_{k}+\varepsilon_{ijk}$$

where *Yij* is the dependent variable, *μ* is the overall mean, $\beta_{j}$ is the fixed effect of the continuous glucose meter, $\gamma_{k}$ is the random effect of animal and $\varepsilon_{ijk}$ is the residual error.

An analysis of variance (ANOVA) was performed on the model and significance level was set as *p* < 0.05. Two models were tested, for model 1 we investigated the CGM readings during the glucose infusion; therefore, whether the CGM could detect an increase in GC associated with exogenous manipulation of animal blood GC. In Model 2 we investigated the CGM reading after the glucose infusion; therefore, whether the CGM could detect a decrease in GC associated with the animal’s physiological glucose clearing mechanisms. For model evaluation, the standard residual error variance (σˆe) and the concordance correlation coefficient (CCC) were used.

To compare the results of all measurement methods, another linear mixed effects model was performed with glucose concentration and type of measurement (glucometer, CGM sensor, and laboratory assay) as fixed effects and animal as a random effect. An ANOVA was performed on the model to identify significance (*p* < 0.05) between the measurement types. Additionally, estimated marginal means (EMM) were evaluated for each measurement type. Because one animal appeared to be an outlier while investigating random intercepts, that animal was removed, and the linear mixed effects model and subsequent ANOVA and EMM analyses were performed again with all three measurement methods.

## 3 Results

**Figure 1:**
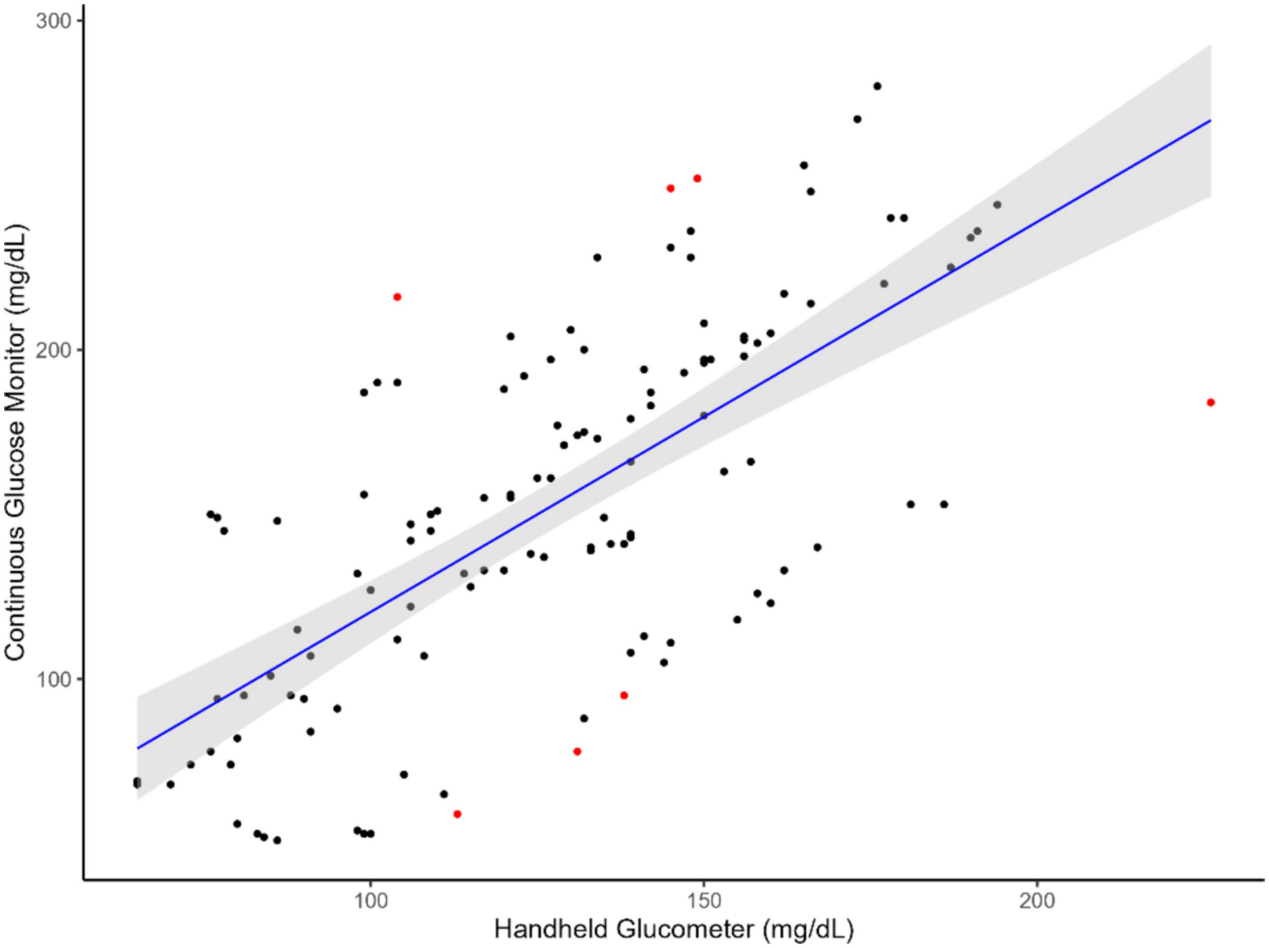
Relationship between blood glucose measurements obtained from the CGM and handheld glucometer during glucose infusion. The blue line represents the regression line of best fit, with the green shaded area indicating the 95% confidence interval. Red dots highlight identified outliers.

**Figure 2:**
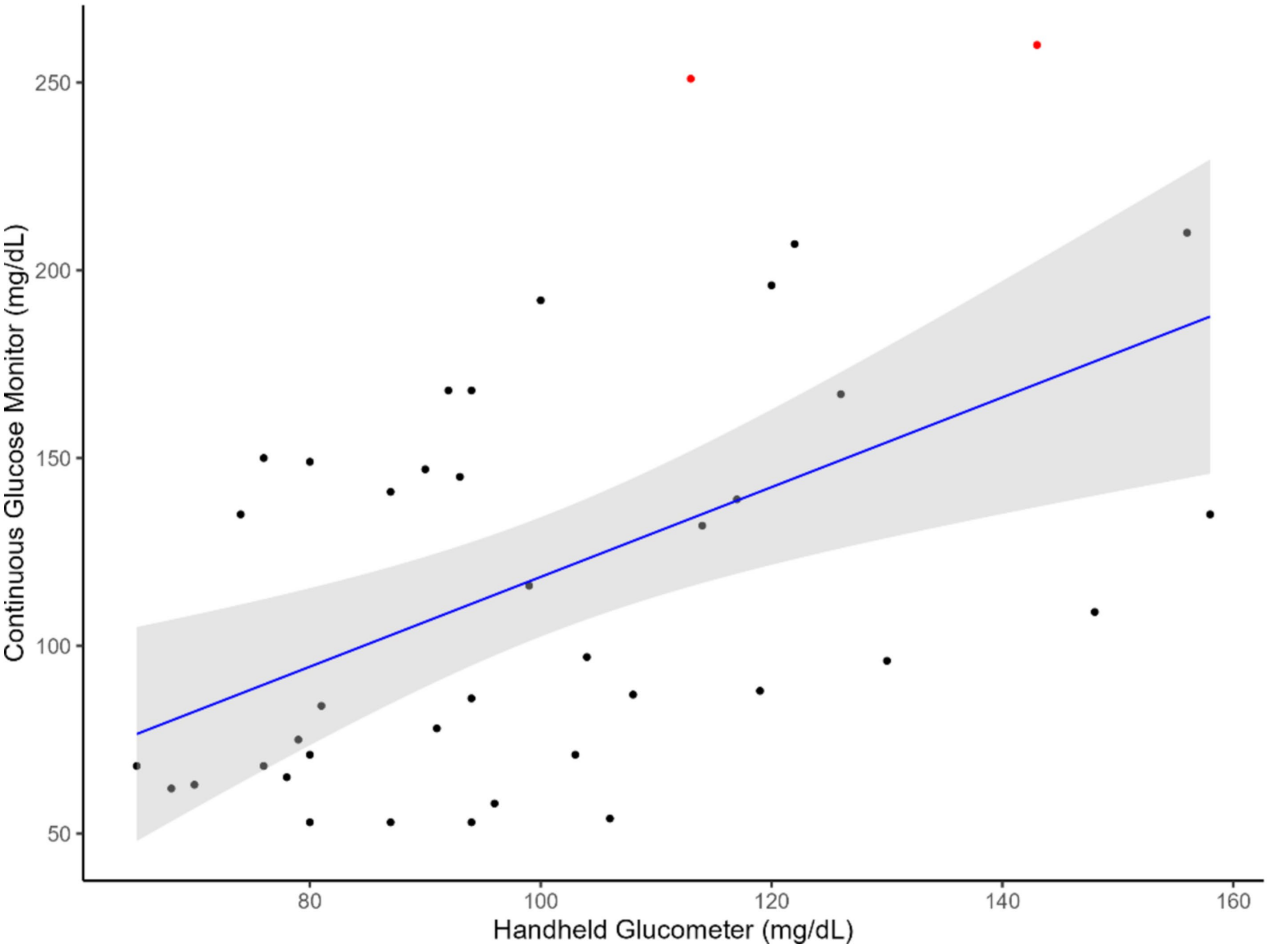
Relationship between blood glucose measurements obtained from the CGM and handheld glucometer after glucose infusion. The blue line represents the regression line of best fit, with the green shaded area indicating the 95% confidence interval. Red dots highlight identified outliers.

The results indicated that CGM followed a linear relationship (Figures 1, 2) with the handheld glucometer both during the infusion (*p* = 0.0003, Table 1) and after the infusion (*p* = 0.006, Table 1). The evaluation of overall accuracy and residual error in our study was based on the CCC and residual error variance obtained from two mixed models developed. The results showed that both models had a high CCC (0.91 and 0.94 for model 1 and 2, respectively) and low sigma error (13.2 and 7.9, for model 1 and 2, respectively). This indicates that the models were effective in explaining blood CG and there was a high accuracy in measuring an increase or decrease using CGM, with low sigma error indicating minimal deviation from the CGM and the handheld glucometer.

**Table 1:** *p*-values of model 1 (CGM readings during infusion) and model 2 (CGM readings after infusion) and evaluation for the two models tested.

Item	SEM	*P*-value
Model 1	0.059	0.0003
Model 2	0.092	0.006

Models tested		
Fit statistics	Model 1	Model 2
CCC	0.91	0.94
σˆ_e_	13.27	7.9

CGM, continuous glucose monitor; CCC, concordance correlation coefficient; σˆe, standard deviation for error.

The results comparing the readings from the glucometer, CGM sensor, and the calorimetric assay are presented in Table 2. Glucose concentrations from the CGM and the glucometer did not differ from the calorimetric assay, but they were significantly different from each other (*p* = 0.045). When calculated with the EMM values, there was a 16.1% difference in concentration between the CGM and the glucometer, and a 12.2% difference in concentration between the CGM and the calorimetric assay. After removing an outlier animal from the analysis, glucose concentrations did not differ significantly among the three measurement methods (*p* = 0.715). With the revised EMM of glucose concentrations, there was only a 3.80% difference in concentration between the CGM and the glucometer, and a 2.47% difference in concentration between the CGM and the assay. The difference between the EMM of the concentrations from the glucometer and the calorimetric assay, both of which are considered standard methods of detection, was 6.17%.

**Table 2:** EMM values of glucose concentration for each measurement tested in the study.

Item	CGM (mg/dL)	Glucometer (mg/dL)	Calorimetric Assay (mg/dL)	*P-*value
EMM^1^ with outlier	180^A^	151^B^	158^AB^	0.045
EMM without outlier	158^A^	152^A^	162^A^	0.715

^1^Estimated marginal means. ∗*p* ≤ 0.05. Different letters indicate statistically significant differences between the measurement methods (*p* ≤ 0.05).

## 4 Discussion

This study aimed to evaluate the feasibility of using a CGM designed for humans to measure glucose levels in sheep. The animals were subjected to a hyperglycemic clamp, during which glucose was infused through a jugular catheter. Reading from the CGM sensors were compared with those from a handheld glucometer to assess their ability to detect changes in GC. We hypothesized that the CGM technology could be used to monitor an increase and decrease in GC in sheep, given that the average circulating GC in sheep are generally 42 to 80 mg/dL which is within the minimum detection glucose level of the CGM used in this study (Figures 1, 2). Therefore, employing an established and existing technology such as the CGM, originally designed for humans, would lead to significant cost and resource savings in both research and industry animal monitoring.

A number of studies have investigated the use of CGM sensors (3–6). Wong et al. (5) evaluated the accuracy and correlation of a CGM (DexCom G6) in measuring GC in neonatal foals and compared the results with a glucometer and with laboratory chemistry analysis. The authors reported and moderate CCC (0.59) and a correlation (0.77) between the CGM and the laboratory analysis, suggesting that the CGM is an acceptable method for providing immediate and continuous glucose measurements in neonatal foals. The authors also noted that the CGM provided more accurate measurements than the glucometer, which frequently overestimated blood glucose levels. Our findings demonstrated that the CGM recorded higher GC than the handheld glucometer. Still, it has been reported that a temporal gap may exist between rapid alterations in blood GC and corresponding changes in interstitial blood concentrations, with a delay of 5–10 min observed in both people (16) and animals (13).

The improper placement of CGM can lead to poor sensor performance and durability of the sensor. This was noticed when we performed the comparison between the two CGM and the handhold glucometer with the standard calorimetric laboratory assay. In a recent study, Byrd et al. (6) evaluated the effectiveness of two CGMs (FreeStyle Libre (FSL), Abbott Laboratories and Dexcom G6, Dexcom Inc., San Diego, CA) in measuring GC in dairy cows. While a significant increase in GC was observed after a glucose bolus dose, the accuracy of the FSL when positioned in the ear was found to be inadequate (0.47), despite a high correlation coefficient (0.82) with blood GC measurements obtained via a handheld glucometer. The authors noted difficulties with the durability of the FSL during their experiment, which lasted approximately 48 h, while in our study the CGM endured for approximately 7 days (note that our results were reported only when animals were subjected to jugular catheter). These differences can be explained by the distinct localities the sensors were employed on the animals in the two studies. Additionally, our study utilized the updated version of the FSL system, the FSL 2, which may have contributed to the differences in accuracy and lifespan between the two studies. Furthermore, Byrd et al. (6) reported that the application of the CGM was a straightforward process, while in our study, we found that the sensors were not secure without the use of superglue. In addition to placement challenges, another obstacle to applying CGMs in livestock research is the limited lifespan of each sensor, which lasts only 14 days and would require frequent replacements for extended use.

In addition to the overall analysis, we also explored the individual animal differences. Although we had a small number of animals, there were significant differences associated with animals that influenced the association between CGM and handheld glucometer measurements. This was particularly the case in model 2 (*p* = 0.003), indicating that individual sensors may have unique intercepts and potentially a mean bias in the measurement. Therefore, although the CGM was able to detect changes in glucose concentrations in sheep, it is recommended that the values obtained from these sensors be standardized to ground truth measurements, such as laboratory analysis using plasma samples (e.g., calorimetric assay), before they substitute the traditional methods used in research or clinical contexts. In addition to the significant shifts associated with the different animals, a major limitation of this study is the small sample size, with only four animals used. Increasing the number of animals in future studies would enhance statistical power and help to better contextualize the impacts and a more comprehensive assessment of individual animal variation. As an additional limitation, the CGM was evaluated under controlled research settings with glucose being infused in the animals. Expanding the study to detect glucose changes in response to different types of feed or physiological conditions would enhance our confidence in the capacity of the technology across various situations. If proven effective in different scenarios, CGM could serve as an effective tool to optimize feeding and management practices, and potently reduce the need for invasive procedures such as frequent blood samplings. While it is important to note that the insertion of a small needle is still required to use this technology, the pain is minimal. The opportunities to improve animal welfare with a CGM are numerous. For instance, in a metabolism study that often requires an infusion, the CGM would eliminate the need for a second catheter for continuous blood sampling. Additionally, under normal conditions, it would decrease the frequency of animal restraint needed for blood collection. These shifts would have significant implications in reducing stress and discomfort associated with frequent invasive procedures. With further research and development, CGM could become a valuable tool for optimizing research and improving animal welfare. As technology advances, CGM may become more widely available. Overall, our study contributed to the growing body of literature on CGM in livestock research, and we anticipate further advancements and future applications for animal health and management.

## 5 Conclusion

The available data from this study suggested that CGM devices intended for human use have the potential to detect changes in interstitial glucose concentrations in sheep. While there is a need to consider factors such as precise application and sensor standardization, CGM has the potential to monitor glucose concentrations with no significant difference from other types of measurement. Future standardization efforts should focus on CGM readings against standard laboratory methods, such as glucose colorimetric assays, to establish a baseline for comparison. It is possible that human intended CGM can accurately measure interstitial glucose concentrations in livestock, though more work should be done to evaluate their use across a greater number of animals, species, and manufacturers. Standardizing CGM results with laboratory analysis, such as glucose calorimetric assays, should be further explored before deploying this tool independently in research or clinical contexts for animal health and management.

## Data Availability

The raw data supporting the conclusions of this article will be made available by the authors, without undue reservation.
